# The Dartmouth Atlas of Health Care – bringing health care analyses to health systems, policymakers, and the public

**DOI:** 10.1007/s43999-022-00006-2

**Published:** 2022-07-27

**Authors:** Kristen K. Bronner, David C. Goodman

**Affiliations:** The Dartmouth Institute for Health Policy & Clinical Practice, Geisel School of Medicine at Dartmouth, Williamson Translational Research Building, Level 5, 1 Medical Center Drive, Lebanon, NH 03756 USA

**Keywords:** Unwarranted regional variation, Small area analysis, Dartmouth Atlas

## Abstract

In 1996, the Dartmouth Atlas of Health Care pioneered the dissemination of policy-relevant population-based measurement and analysis that revealed both weaknesses and opportunities in the United States health care system by focusing on regional and hospital variation in utilization, quality, and costs. Built on a growing foundation of peer-reviewed research, the Atlas produced more than 40 reports over the next 25 years addressing a wide range of pressing health care problems. The project’s publications and website also provided regional and hospital-specific data to health systems, governmental jurisdictions, health care stakeholders, and the public. The Atlas’ methods and its conceptual framework have been widely disseminated in North America and the United Kingdom, and, more recently, in Europe, South America, Asia, and Oceania. This paper discusses the origins of the Atlas from Dr. John Wennberg’s early studies, the scaling up of data, methods, and policy-relevant findings, and its incorporation into the more general fields of health services research, policy development, and clinical improvement.

## Introduction

In 1996, the Dartmouth Atlas of Health Care pioneered the dissemination of policy-relevant population-based measurement and analysis that revealed both weaknesses and opportunities in the United States health care system by focusing on regional and hospital variation in utilization, quality, and costs. Built on a growing foundation of peer-reviewed research, and with support from the Robert Wood Johnson Foundation (Princeton, New Jersey, USA), the Atlas produced more than 40 reports over the next 25 years addressing a wide range of pressing health care problems. The project’s publications and website also provided regional and hospital-specific data to health systems, governmental jurisdictions, health care stakeholders, and the public. The Atlas’ methods and its conceptual framework have been widely disseminated in North America and the United Kingdom, and, more recently, in Europe, South America, Asia, and Oceania. This paper discusses the origins of the Atlas from Dr. John Wennberg’s early studies, the scaling up of data, methods, and policy-relevant findings, and its incorporation into the more general fields of health services research, policy development, and clinical improvement.

## The context

In the middle to late twentieth century, health care in the U.S. was both highly successful and deeply troubled. Major advances in biomedicine—including new surgical techniques, medical treatments, and prescription drugs—held great promise for reducing the burden of disease for patients with a host of acute and chronic conditions. However, access to these advanced services was uneven, frequently expensive, and often only available in hospitals, which varied widely in accessibility, cost, and quality [1].

The advent of Medicare in 1965 provided insurance for the aged, and in 1966, Medicaid provided increasingly comprehensive coverage for the low-income population. At the same time, Congress sought to speed the dissemination of biomedical discoveries. The basis for a new initiative, Regional Medical Programs (RMPs), was the 1964 report of the President’s Commission on Heart Disease, Cancer and Stroke, which recommended a program “designed to bring together the best in medical care and the best in medical research, region by region across the nation… It will make available to every doctor in the country the newest and most effective diagnostic methods and the most promising methods of treatment. It will, in effect, link every private doctor and every community hospital to a national—and indeed worldwide—network transmitting the newest and best in health service” [2]. In 1965, Congress authorized and appropriated federal funding for the creation of RMPs to develop these networks [3], and Dr. John “Jack” Wennberg became the director of the Northern New England RMP, based at the University of Vermont in Burlington.

## Small area analysis and the origins of the Dartmouth Atlas

Much of the basis of the Dartmouth Atlas of Health Care—both fundamental themes and methodology—originated in Dr. Wennberg’s early studies in New England, first in Vermont, and later in the states of Maine and Rhode Island and the cities of Boston, Massachusetts and New Haven, Connecticut.

### A novel approach

Charged with designing a plan to meet the health needs of the population of Vermont, Dr. Wennberg relied on his training in epidemiology “to provide population-based information about the distribution of health care resources and the utilization of services among Vermont communities” [4]. In their first small area analysis, published in *Science* in 1973, Dr. Wennberg and his co-author Dr. Alan Gittelsohn defined 13 “hospital service areas” in Vermont, grouping towns together according to the hospital most frequently used by the towns’ residents, and assembled data from various sources—including hospital discharge abstracts, surveys of hospitals and health care agencies, and Medicare Part B reimbursement claims (medical claims for inpatient clinician and outpatient professional services)—to characterize the medical care experience of the population of each area [5]. This approach foreshadowed the later development in the 1990s of hospital service areas and hospital referral regions nationwide by the Dartmouth Atlas project as the areal units for measuring and comparing per capita resource inputs, utilization, and outcomes across health care market areas.

### Emerging themes

Several of the key premises that form the core of the Dartmouth Atlas project are supported by Dr. Wennberg’s early research and have been replicated across varied populations using many different research designs.

- *Much of the observed variation in health care across areas is “unwarranted*.” The expectation for the first study of Vermont, a small, largely rural, demographically homogeneous state, was that small area analysis would reveal widespread underservice. Instead, the researchers found large variations in most of the measures they studied—hospital and nursing home admissions, surgical procedures, health care resources, and expenditures per capita—across the hospital service areas. These variations did not appear to reflect differences in the needs of the population, but rather professional uncertainty about when to use a particular kind of service, which in turn was linked to a lack of clinical trials providing evidence regarding the outcomes of treatment. In other words, the variations appeared to be “unwarranted”; not based on either patient need or informed demand, or on evidence regarding effectiveness [5].
- *In the absence of evidence supporting a clearly superior intervention, variation in care often reflects physician preferences without accounting for the preferences of informed patients.* Follow-up studies in Maine verified that the unwarranted variation phenomenon was not unique to Vermont [6] and led to a collaboration with local physicians in Maine to attempt to understand the decision-making processes that led to the large differences in surgery rates between communities. The group uncovered two competing theories on the reasons for recommending surgery: the preventive theory, or operating early in the course of an illness to prevent poor outcomes in the future; and the quality of life theory, or controlling symptoms and improving wellbeing while avoiding possibly unnecessary surgery. While the collaboration was focused on treatment options for benign prostatic hyperplasia (including watchful waiting and open or transurethral prostatectomy), subsequent research found similarly high variation for many surgical procedures that could not be explained by patient-related factors. These included tonsillectomy, hysterectomy, hip replacement, carotid endarterectomy, and back surgery, to name a few [7, 8]. This work brings to light the importance of involving the patient in decision-making for “preference-sensitive” conditions—those for which more than one treatment option exists and there are significant trade-offs affecting quality and/or length of life—especially in situations where evidence regarding outcomes is scarce and surgical mortality rates might be high [9].
- *Local supply of resources is an important driver of utilization.* The Vermont study noted the correlations between the differences in utilization rates and the local supply of physicians providing a given service. Later research comparing academic medical centers in the cities of Boston (Harvard, Tufts, and Boston University hospitals) and New Haven (Yale-New Haven Hospital) supported and expanded upon this finding. There was widespread agreement that these prestigious academic medical centers delivered high-quality care based on the best available scientific evidence. Despite this consensus, Dr. Wennberg revealed in a 1984 paper in *Health Affairs* that physicians in Boston used twice as many resources in caring for their patients than did those in New Haven [10]. A subsequent study revealed that “[m]ost of the extra beds [used in Boston] were invested in higher admission rates for medical conditions in which the decision to admit can be discretionary” [11]. The idea that the availability of extra beds in Boston might, in and of itself, lead to an increase in discretionary admissions was not a new one; Milton Roemer had observed as early as the 1960s that available hospital beds will tend to be used, no matter how many beds there are [12]. The research group came to call this type of care—care whose frequency of use is determined not by scientific evidence, or even local medical opinion, but by the availability of resources—“supply-sensitive.”
- *More health care might not be better.* One important outcome—age-adjusted mortality—was not better for Vermont residents living in areas with more health care resources and higher spending for hospital and physician services. There was also virtually no difference in age, sex, and race standardized mortality rates among the residents of Boston and New Haven [13]. The mechanisms by which more medical care has the potential to lead to harm include lower diagnostic thresholds leading to more labeling of people as having a disease; more treatment of clinically detected conditions that might never have caused harm; and more distraction and potential for adverse events and mistakes [14]. Unnecessary treatment has potentially negative consequences for both patients (e.g., iatrogenic illness, poor outcomes) and for the larger health care system (e.g., waste, inequity in spending and resource allocation). In the face of these possibilities, Dr. Wennberg and his colleagues’ studies demonstrated that that more health care is not necessarily better, and, in some instances, it is worse.

## The first Dartmouth Atlas

The election of President Bill Clinton in 1992 gave rise to the expectation of health care reform, and Dr. Wennberg’s Dartmouth research team anticipated the need for accurate, objective data demonstrating the impact of the new legislation on the U.S. health care system, both as a whole and at the regional level. Using national Medicare datasets and the methods of small area analysis developed during the 1970s and 1980s seemed ideal for this undertaking. With the support of the Robert Wood Johnson Foundation, work began to create health care market areas for the nation and to define a set of indicators—including spending, supply, and utilization variables—that could be measured before and after the implementation of health care reform.

### Definition of Atlas regions

**Fig. 1:**
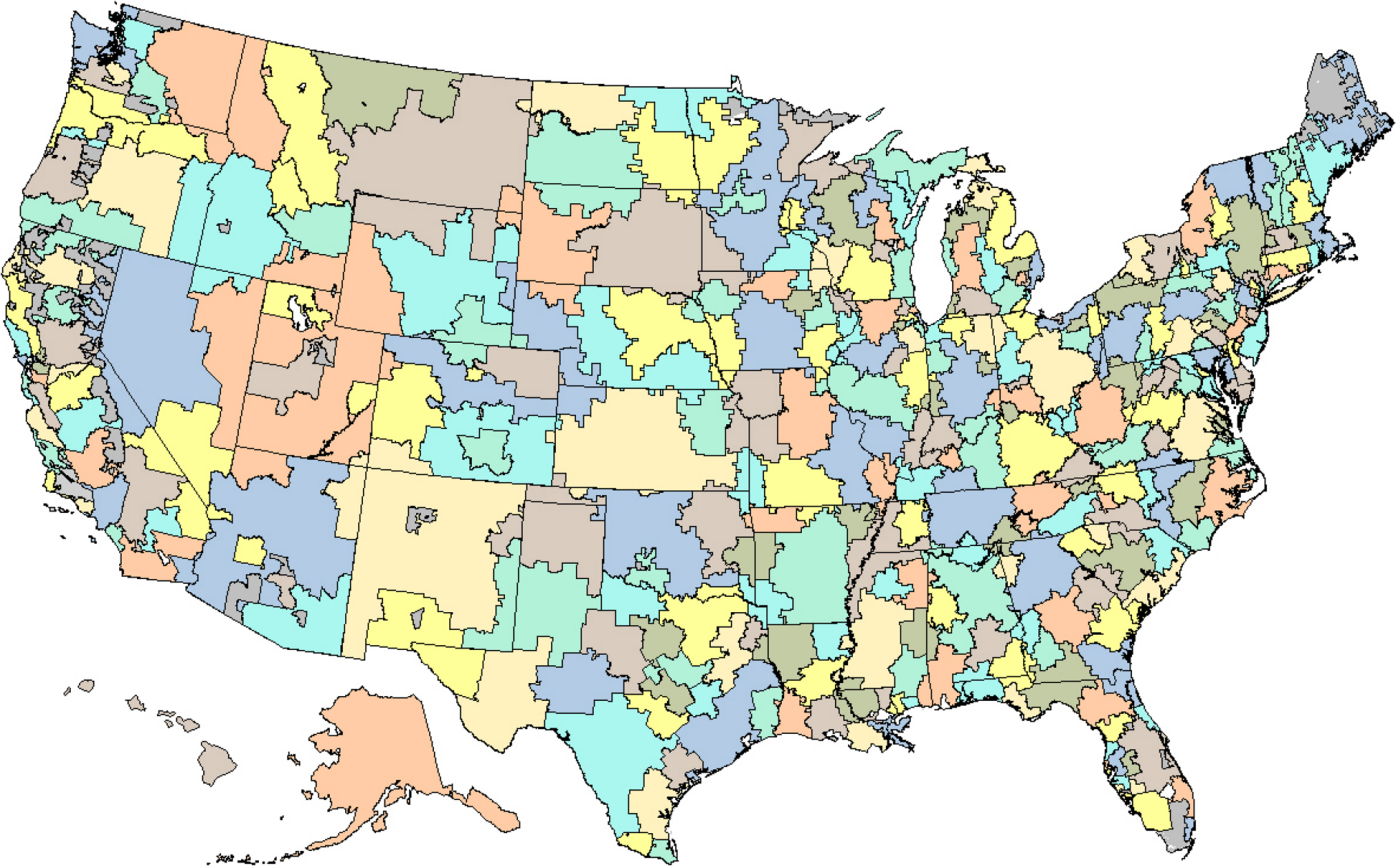
Dartmouth Atlas hospital referral regions. The Dartmouth Atlas of Health Care developed 306 hospital referral regions, or regional health care markets for tertiary medical care, in the early 1990s. Each region contained at least one hospital that performed major cardiovascular procedures and neurosurgery. Hospital referral regions were defined by assigning hospital service areas to the region where the greatest proportion of major cardiovascular procedures were performed, with minor modifications to achieve geographic contiguity, a minimum population size of 120,000, and a high localization index; most of the care delivered to patients living in the region occurred within the region

The team defined 3436 hospital service areas (HSAs) by assigning ZIP (postal) codes to the hospital area where the greatest proportion of their Medicare residents were hospitalized. These areas were further grouped into 306 “hospital referral regions” (HRRs) in order to create larger, self-contained markets for tertiary medical care, such as major cardiovascular surgery and neurosurgery (Fig. 1). The smaller HSAs provided greater population and provider specificity, particularly when the area contained only a single hospital. This was rarely the case in urban settings, where populations often crossed HSA boundaries for inpatient care. Focusing on the larger HRRs reduced the likelihood that a significant proportion of the resources and services used to care for patients living there were being delivered at facilities outside the region.

### Data sources

Research data available from Medicare, the federal health insurance plan that covers nearly every U.S. resident age 65 and over, was determined to be the best source of data for the project; while this would restrict the study to elderly patients, there was—and remains today—no counterpart to this database for the general population. Medicare claims data, along with data on the supply of hospital beds, employees, and nurses from the American Hospital Association and on the physician workforce from the American Medical Association, were the foundation of the first Dartmouth Atlas of Health Care.

### Atlas themes

Though the Clinton health plan failed to pass, the first Atlas was nevertheless published in 1996 [15]. The national edition focused on data at the HRR level and was accompanied by nine regional volumes reporting local data for HSAs. This Atlas brought together and expanded upon the themes of unwarranted variation developed in earlier variation studies. The question of “which rate is right?” remained central, for both the allocation of resources and the utilization of care. The effect of capacity on utilization was explored in depth, demonstrating the relationship between a higher supply of resources and greater use of care—especially supply-sensitive care—at the national level. For preference-sensitive surgery, the Atlas uncovered marked variation and advocated for the patient to be fully informed and empowered to make the choice to undergo or forego a procedure in situations where the available treatment options have varying risks and benefits. Variation in the use—and frequent underuse—of medical care services that have been shown to be effective at improving outcomes, such as mammography for women over age 50, was also presented. Ironically, many of the regions with high utilization of supply-sensitive care had low rates of use of effective care. Attention was also given to the “is more better?” question: whether outcomes—primarily mortality—were better in regions with high capacity and high costs. Finally, issues of equity and fairness were raised, as higher health care costs in high-intensity regions meant the transfer of federal health care dollars from low-use to high-use regions; in effect, residents of regions with lower utilization subsidize the care of patients in high-use ones, despite the lack of evidence that medical needs or patient preferences differ significantly across regions.

**Fig. 2:**
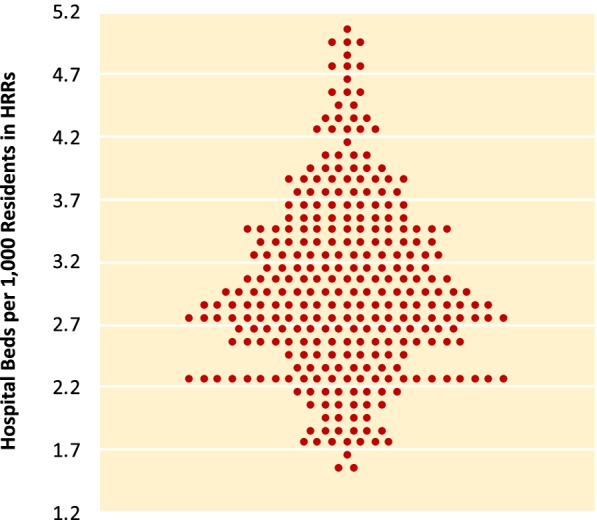
Distribution graph. The Dartmouth Atlas of Health Care developed the distribution graph to show the variation of a particular measure across health care market areas or hospitals. It shows both the overall variation and whether it is caused by a few outliers or is widespread across the studied population. In this example, the dispersion is not caused by outliers, but rather is widespread. Each dot represents one of 306 hospital referral regions in the United States. From Wennberg JE, Cooper MM, Bubolz TA, et al. The Dartmouth Atlas of Health Care 1998. Chicago, IL: American Hospital Publishing, Inc.; 1998

The first Dartmouth Atlas was largely descriptive, using relatively simple methods of indirect adjustment for population demographics and, for Medicare reimbursement measures, regional differences in prices. It introduced the distribution graph, which shows both the overall variation for the measure in question and whether this variation is caused by a few outliers or is pervasive and widespread across regions (Fig. 2), and which has become a signature feature of the Dartmouth Atlas. Future reports would build upon the themes synthesized in the first Atlas, adding new measures and developing more sophisticated methods, while continuing to return to the core questions. Which rate is right? How much is enough? And what is fair?

## Expanding the scope

**Fig. 3:**
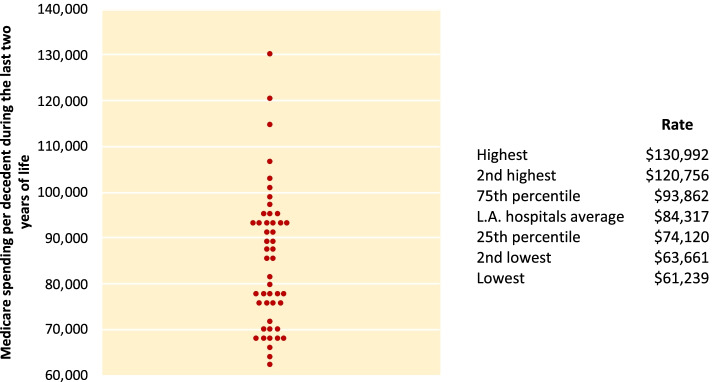
Medicare spending per decedent during the last 2 years of life for patients with at least one of nine chronic conditions among Los Angeles hospitals (deaths occurring 2001–05). Each dot represents one of 48 hospitals in the Los Angeles hospital referral region. The table accompanying the figure gives the rates for the two hospitals with the highest spending; the hospital located at the 75th percentile; the average for all included Los Angeles hospitals; the hospital at the 25th percentile; and the two lowest Los Angeles hospitals. Medicare reimbursement rates for the care of patients who were in their last 2 years of life varied more than twofold across Los Angeles. Adapted from Wennberg JE, Fisher ES, Goodman DC, Skinner JS, Bronner KK, Sharp SM. Tracking the Care of Patients with Severe Chronic Illness: The Dartmouth Atlas of Health Care 2008. Lebanon, NH: The Dartmouth Institute for Health Policy & Clinical Practice; 2008

In the two decades following the publication of the initial Dartmouth Atlas of Health Care, the research team at Dartmouth, along with many outside collaborators, has produced almost 50 additional reports of varying length and breadth (Table 1). Some have focused on specific clinical topics, including but not limited to cardiovascular and vascular disease, musculoskeletal conditions, diabetes, obesity, cancer, and neonatal intensive care. Others have taken advantage of more recently available Medicare claims files to expand both the populations and the topics under study. For example, the availability of 100% samples of all claims for Medicare beneficiaries, including clinician claims, has allowed robust measurement of utilization and spending at the hospital level. Hospital-specific data is more actionable and demonstrates that, even within extremely localized areas such as one city, variation across providers can be quite high (Fig. 3) [16]. In another example, the release of the Medicare Part D prescription drug program’s claims database allowed a deep exploration of prescription pharmaceutical use by Medicare beneficiaries [17].

**Table 1:** Dartmouth Atlas publications

Title	First Author	Year	Population(s) studied
The Dartmouth Atlas of Health Care: National and 9 regional volumes	Wennberg JE	1996	FFS Medicare age 65+ (utilization) U.S. Census population (resources)
The Dartmouth Atlas of Health Care 1998	Wennberg JE	1998	FFS Medicare age 65+ (utilization) U.S. Census population (resources)
The Dartmouth Atlas of Health Care in Pennsylvania	Wennberg JE	1998	Pennsylvania residents (hospitalizations) FFS Medicare age 65+ (Medicare utilization & reimbursements) U.S. Census population (resources)
The Dartmouth Atlas of Cardiovascular Health Care	Wennberg DE	1999	FFS Medicare age 65+ (utilization) U.S. Census population (resources)
The Quality of Medical Care in the United States: A Report on the Medicare Program: The Dartmouth Atlas of Health Care 1999	Wennberg JE	1999	FFS Medicare age 65+ (utilization) U.S. Census population (resources)
The Dartmouth Atlas of Vascular Health Care	Cronenwett JL	2000	FFS Medicare age 65+ (utilization) U.S. Census population (resources)
The Dartmouth Atlas of Musculoskeletal Health Care	Weinstein JN	2000	FFS Medicare age 65+ (utilization) U.S. Census population (resources)
The Dartmouth Atlas of Health Care in Virginia	Wennberg JE	2000	Virginia residents (hospitalizations) FFS Medicare age 65+ (Medicare utilization & reimbursements) U.S. Census population (resources)
The Dartmouth Atlas of Health Care in Michigan	Wennberg JE	2000	Enrollees of Blue Cross Blue Shield of Michigan (utilization, prescription drugs) FFS Medicare age 65+ (Medicare utilization & reimbursements) U.S. Census population (resources)
Cardiac Surgery		2005	FFS Medicare age 65+
Spine Surgery		2006	FFS Medicare age 65+
Trends and Regional Variations in Abdominal Aortic Aneurysm Repair		2006	FFS Medicare age 65+
The Care of Patients with Severe Chronic Illness: A Report on the Medicare Program: The Dartmouth Atlas of Health Care 2006	Wennberg JE	2006	FFS Medicare decedents age 67+
An Agenda for Change: Improving Quality and Curbing Health Care Spending: Opportunities for the Congress and the Obama Administration	Wennberg JE	2008	FFS Medicare age 65+
Disparities in Health and Health Care Among Medicare Beneficiaries	Fisher ES	2008	FFS Medicare age 65+
Regional and Racial Variation in Health Care Among Medicare Beneficiaries	Fisher ES	2008	FFS Medicare age 65+
Geography Is Destiny: Differences in Health Care Among Medicare Beneficiaries in the United States and California	Fisher ES	2008	FFS Medicare age 65+
Tracking the Care of Patients with Severe Chronic Illness: The Dartmouth Atlas of Health Care 2008	Wennberg JE	2008	FFS Medicare decedents age 67+
Hospital and Physician Capacity Update	Goodman DC	2009	U.S. Census population
Health Care Spending, Quality, and Outcomes: More Isn’t Always Better	Fisher ES	2009	FFS Medicare age 65+
The Policy Implications of Variations in Medicare Spending Growth	Fisher ES	2009	FFS Medicare age 65+
Regional and Racial Variation in Primary Care and the Quality of Care Among Medicare Beneficiaries	Goodman DC	2010	FFS Medicare age 65+
Trends and Regional Variation in Hip, Knee, and Shoulder Replacement	Fisher ES	2010	FFS Medicare age 65+
Trends and Regional Variation in Carotid Revascularization	Goodney PP	2010	FFS Medicare age 65+
Quality of End-of-Life Cancer Care for Medicare Beneficiaries: Regional and Hospital-Specific Analyses	Goodman DC	2010	FFS Medicare decedents age 67+
After Hospitalization: A Dartmouth Atlas Report on Post-Acute Care for Medicare Beneficiaries	Goodman DC	2011	FFS Medicare age 65+ (hospitalized)
A New Series of Medicare Expenditure Measures by Hospital Referral Region: 2003–2008	Skinner JS	2011	FFS Medicare age 65+
Trends and Variation in End-of-Life Care for Medicare Beneficiaries with Severe Chronic Illness	Goodman DC	2011	FFS Medicare decedents age 67+
Improving Patient Decision-Making in Health Care: A 2011 Dartmouth Atlas Report Highlighting Minnesota 9 regional volumes in 2012	Brownlee S	2011	FFS Medicare age 65+
What Kind of Physician Will You Be? Variation in Health Care and Its Importance for Residency Training	Arora A	2012	FFS Medicare age 65+
The Dartmouth Atlas of Children’s Health Care in Northern New England	Goodman DC	2013	Commercially & Medicaid-insured children < age 18
The Dartmouth Atlas of Medicare Prescription Drug Use	Munson JC	2013	40% enrollees in Medicare Part D
Trends in Cancer Care Near the End of Life: A Dartmouth Atlas of Health Care Brief	Goodman DC	2013	FFS Medicare decedents age 67+
Tracking Improvement in the Care of Chronically Ill Patients: A Dartmouth Atlas Brief on Medicare Beneficiaries Near the End of Life	Goodman DC	2013	FFS Medicare decedents age 67+
Measuring Up? End-of-Life Cancer Care in California	Brownlee S	2013	FFS Medicare decedents age 67+
End-of-Life Care in California: You Don’t Always Get What You Want	Brownlee S	2013	FFS Medicare decedents age 67+
Variation in the Care of Surgical Conditions: Cerebral Aneurysms	Bekelis K	2014	FFS Medicare age 65+
Variation in the Care of Surgical Conditions: Prostate Cancer	Hyams ES	2014	Male FFS Medicare age 65+
Variation in the Care of Surgical Conditions: End-Stage Renal Disease	Zarkowsky D	2014	FFS Medicare age 18 + Enrollees in ESRD program
Variation in the Care of Surgical Conditions: Spinal Stenosis	Martin BI	2014	FFS Medicare age 65+
Variation in the Care of Surgical Conditions: Diabetes and Peripheral Arterial Disease	Goodney PP	2014	FFS Medicare age 65+
Variation in the Care of Surgical Conditions: Obesity	Reames BN	2014	FFS Medicare age 65+
Our Parents, Ourselves: Health Care for an Aging Population	Bynum JPW	2016	FFS Medicare age 65+
The Dartmouth Atlas of Neonatal Intensive Care	Goodman DC	2019	U.S. total birth cohort Commercially & Medicaid-insured newborns in Texas Newborns in Norway
End of Life Cancer Care	Barnato A	2020	FFS Medicare decedents age 66+
The Dartmouth Atlas of Health Care: 2018 Data Update	Bronner KK	2021	FFS Medicare age 65+

*FFS* fee for service, *ESRD* end-stage renal disease

The Atlas project has also taken advantage of databases other than Medicare to report on variation in non-elderly populations, including commercial insurance claims, state and regional all-payer databases, and Medicaid (the federal insurance program covering low-income residents) claims. These reports have shown that unwarranted variation is by no means a phenomenon that occurs only among the elderly; if anything, variation is higher outside of the context of Medicare’s relatively uniform coverage and regulated environment. Efforts to uncover unmeasured confounders that might indicate that observed variations are warranted have, time and again, failed to produce evidence to refute some of Dr. Wennberg’s earliest assertions: capacity, once built, will be used; and local medical culture, rather than patient needs or preferences, is a significant driver of utilization.

## The role of public reporting

The Centers for Medicare and Medicaid Services (CMS) actively promotes the use of Medicare datasets for research and also allows the identification of hospitals by name. In 2006, Dr. Wennberg and colleagues published the first Atlas with hospital-specific measures for patients with severe chronic illness. Because the possibility of confounding by patient selection is much higher for hospitals than regions, the Atlas team developed a novel method of risk adjustment by defining decedent cohorts with chronic illness and then measuring utilization in the last 6–24 months of life. Patients in these cohorts had at least one of eleven serious chronic conditions shown to have a high probability of death in the hospital; in turn, the prevalence of each of these conditions, determined by the final hospital admission prior to death, was used to adjust for difference in case mix across hospital cohorts. Utilization rates were also adjusted for patient age, sex, and race [18, 19]. At both the regional and hospital level, the patterns of care for patients in the last 24 months of life are highly correlated with those for chronic illness cohorts measured prospectively. Despite this strict risk adjustment, hospital-specific measures of end-of-life inpatient and intensive care unit utilization, imaging, testing, and physician visits varied markedly. This and subsequent hospital-specific reports received high levels of stakeholder and media attention because of the questions they raised about the intensity and quality of care delivered at well-known hospitals. Today, public reporting of hospital performance is routinely available from CMS and other health care payers.

## The Atlas research engine

The use of non-traditional methods of communicating research findings, such as the Dartmouth Atlas, has raised legitimate questions regarding the validity of the methods and the reported findings. The Dartmouth Atlas team is led and staffed by academic researchers, and the Atlas findings are supported by several hundred peer-reviewed and published papers [20]. This vigorous research activity was continuously supported by the primary funder of the Atlas, the Robert Wood Johnson Foundation, and numerous other sources of research funding (past and current) including the National Institutes of Health, the Health Resources and Services Administration, the Agency for Healthcare Research and Quality, the Charles H. Hood Foundation, the Anthem Foundation, and the California HealthCare Foundation. The project continues to support non-Atlas researchers by freely offering on its website HSA, HRR, and hospital-level datasets that span the period 1992–2019.

## Developing policy and clinical remedies

The Dartmouth Atlas, along with the rest of Dr. Wennberg’s variation studies, demonstrated the existence of unwarranted variation beyond any reasonable doubt. As early as the mid-1970s, policy proposals were under development to address the myriad issues causing these variations: scientific uncertainty regarding the risks and benefits of common surgical procedures and the resulting inability of patients to make fully informed choices [21]; the lack of an infrastructure to support shared decision-making, even were the evidence available [22]; the lack of a systematic process to measure the performance of hospital markets against benchmarks for efficient utilization of resources [10]; and payment models that lead to disorganized, fragmented care and reward the provision of high-cost services without regard for outcomes [23].

Federal legislative remedies have met with mixed success. Seemingly benign propositions—like the idea that outcomes research should be funded in order to establish the scientific basis and practice guidelines for medical care—often fell victim to the American political process, with provisions that might actually impact health care practice being removed in order to appease constituencies who benefit from the status quo [24, 25]. Despite these lobbying efforts, this remains an area of active and productive clinically-relevant research. Other legislation established Medicare demonstration projects to tackle the various causes of unwarranted variation, notably Section 646 of the Medicare Prescription Drug, Improvement, and Modernization Act of 2003. This provision authorized CMS to create “Comprehensive Centers for Medical Excellence” to “develop and test a comprehensive approach for reducing medical errors, involving patients in the choice of preference-sensitive care, and improving management of chronic illness… [d]emonstration projects could develop new payment methodologies that will promote the integration of the various components of the health care delivery system and result in shared savings that can be used to further improve care” [26]. Explicitly based on Dr. Wennberg’s body of research, this provision provided an important foundation for the next round of health reform legislation.

## Heightening the impact

The election of President Barack Obama in 2007 provided another opportunity for national health care reform. In anticipation, the Dartmouth research team summarized the major findings and policy implications from its decades of research on unwarranted variations in a white paper, “An Agenda for Change: Improving Quality and Curbing Health Care Spending. Opportunities for Congress and the Obama Administration” [27]. The report identified the following shortcomings in the U.S. health care system:

- “disorganized, poorly coordinated, and inefficient care that results in the underuse of effective medical interventions and the overuse of physician visits, consultations, hospitalizations and stays in intensive care units, particularly in treating chronic illness;
- clinical decisions that fail to adequately take patient preferences into account, resulting in unnecessary, unwanted elective surgery;
- poor clinical science;
- workforce policies that have resulted in an undersupply of primary care physicians, and an oversupply of physician specialists; and
- insurance markets that are ill equipped to address unwarranted geographic variation in health care delivery because the prices of premiums in any given region are not closely linked to the local cost of medical care.”

The report advocated for the federal government to develop a comprehensive approach to creating organized systems of care, establish a physician workforce policy that supports the goals of organized care, require informed patient choice and shared decision-making, and fund research to provide the scientific basis for cost-effective care.

The Patient Protection and Affordable Care Act (or ACA) passed in 2010. While much of the focus has been on providing health insurance coverage for the uninsured and reforming the insurance market, it also included provisions targeting the shortcomings identified in the white paper. New models of health care delivery are intended to provide financial incentives for delivering organized care, including the creation of “accountable care organizations” to take responsibility for the quality and costs of care for their patient populations; primary care “medical homes” to promote care management rather than high-priced procedures; and bundled payments consolidating all of the services required for an episode of care—physician, hospital, and post-acute services—into a single payment in order to encourage the various providers to work together [28]. The Patient-Centered Outcomes Research Institute was created, with a mandate to “improve the quality and relevance of evidence available to help patients, caregivers, clinicians, employers, insurers, and policy makers make better-informed health decisions” [29]. The ACA also established a national health workforce commission, though it never received funding [30].

Other priorities outlined in the white paper and other policy studies did not fare as well. Shared decision-making, while promoted in various provisions, is not required. Other than those provided by the Dartmouth Atlas, routine reports monitoring the performance of local health care systems and comparing them to benchmarks of quality and efficiency have yet to emerge. Limits on resource allocation and spending, including transfer payments from more efficient, low-use regions to high-use ones, have also not been enacted. Though the ACA attempted to address some of the problems raised by Dr. Wennberg and the Dartmouth research team, the federal government has not, to date, developed a comprehensive plan to take on the fundamental drivers of unwarranted variation in health care. Nevertheless, both clinical improvement and innovation in financing and service delivery have been stimulated and guided by the Atlas and associated research.

## International dimensions

As the Atlas project’s activities matured in the past 10 years, and its ideas and methods became increasing incorporated into the “mainstream” of U.S. health care research, policy development, and innovation, Atlas faculty looked beyond the U.S. borders at efforts to measure population-based health care in other high-income countries. In 2010, with the exception of Canada and the United Kingdom, there were very few attempts to measure health care at the scale of delivery—small areas for primary and hospital care, and larger regions for tertiary care [31]. Measurement at the hospital level was rare, and outside the U.K and in Germany, there was virtually no public reporting. To assist in accelerating the development of the field—in the publication of both Atlases and foundational research—Dr./Prof. David Goodman of Dartmouth and Prof. Gwyn Bevan of the London School of Economics convened a new research forum, the Wennberg International Collaborative (https://wennbergcollaborative.org) that has held regular research and policy meetings that have attracted attendees from every continent. This collaborative continues to be active in developing and disseminating policy-relevant population-based research within and across countries.

## Today and tomorrow

Nearly 60 years after the publication of Dr. Wennberg’s original paper in *Science*, the paradox of success and troubling problems continues in U.S. health care; great leaps forward have been made in managing and curing disease, but these advances are often expensive and not necessarily accessible to those with the greatest need. Moreover, significant amounts are still spent on care that patients, if fully informed, might neither need nor want. But there are reasons for optimism. Ideas that were revolutionary when Dr. Wennberg began his work—that high expenditures and utilization do not necessarily indicate medical need, that the patient should play a central role in decision-making, and that spending more and doing more do not always lead to better health outcomes—are widely accepted in the health policy debate. Research teams in other countries have begun to examine regional variations in their own market areas and to address issues specific to their health systems.

The Dartmouth Atlas continues to produce its annual database, and future reports are planned that will explore racial and ethnic disparities in health care as well as the COVID-19 pandemic. As these reports will continue to demonstrate, the influence of geography in health and health care has only become more important.

## Summary

The Dartmouth Atlas of Health Care synthesizes nearly five decades of research on the causes and consequences of geographic and hospital variations in health care delivery in the United States. These variations are often unwarranted, reflecting neither patient need nor demand, and are instead driven by external factors such as physician preferences and the local supply of resources. Unnecessary treatment has negative consequences for patients, populations, and health care systems in the form of potential adverse outcomes, waste, and inequity. The project has impacted not only the health care debate in the United States, but also in other countries where similar efforts to measure health care across populations and providers are now under way.
